# Adults with a history of melanoma have higher odds of laryngeal cancer in the United States

**DOI:** 10.1016/j.jdin.2025.12.006

**Published:** 2025-12-24

**Authors:** Aya Youssef, Yeon Joo Kim, Brandon Smith, Joe K. Tung

**Affiliations:** a University of Pittsburgh School of Medicine, Pittsburgh, Pennsylvania; b Department of Dermatology, University of Pittsburgh Medical Center, Pittsburgh, Pennsylvania

**Keywords:** cancer association, general dermatology, melanoma, National Health Interview Survey, NHIS, prevalence

*To the Editor:* Melanoma is the fifth most common cancer in the United States, with its incidence increasing at an average rate of 1.2% annually over the past decade.^1^ While prior research (1973-2006) found that individuals diagnosed with melanoma are at an increased risk for subsequent primary malignancies, recent analysis on melanoma and its associated malignancies is limited.^2^

To address this gap, we analyzed the 2021-2023 National Health Interview Survey, a public cross-sectional survey database that provides sampling weights to create a US representative population. Our sample consists of 29,486 adults (aged 18 years and more), and the prevalence of melanoma was calculated to be 0.89% (972 unweighted cases). Chi-squared and subpopulation logistic regression models were performed using STATA/MP 17.0 (StataCorp).

Our results show that individuals diagnosed with melanoma are significantly more likely to be non-Hispanic White, male, and aged more than 60 years (Table I). There is no statistically significant difference in melanoma prevalence based on body mass index or between different geographic regions (Northeast, Midwest, South, and West). After controlling for age and sex, logistic regression analysis reveals 4 cancers significantly associated with cutaneous melanoma: bladder cancer (adjusted odds ratio: 2.31, 95% confidence interval [CI]: 1.41-4.06, *P* = .004), breast cancer (adjusted odds ratio: 1.49, 95% CI: 1.03-2.17, *P* = .03), laryngeal cancer (adjusted odds ratio: 9.97, 95% CI: 2.64-37.58, *P* = .001), and liver cancer (adjusted odds ratio: 3.79, 95% CI: 1.25-11.51, *P* = .02) (Table II). After running a Bonferroni correction to test for multiple comparisons, only laryngeal cancer was found to be significantly associated with cutaneous melanoma.

**Table I:** Characteristics of patients with skin melanoma compared to those without skin melanoma in NHIS 2021-2023

Characteristic	Weighted %∗ (95% CI)	Weighted %∗ (95% CI)	*P* value
	Melanoma of skin†	No melanoma of skin	
Prevalence	0.89 (0.82-0.97)	99.11 (99.03-99.18)	**N/A**
Sex			
Male	54.0 (50.5-57.5)	48.5 (48.2-48.9)	**.003**
Female	46.0 (42.5-49.5)	51.5 (51.1-51.8)	
Age (y)			
18-30	1.0 (0.4-2.1)	22.1 (21.6-22.6)	**<.0001**
31-40	2.4 (1.6-3.7)	17.4 (17.0-17.7)	
41-50	8.7 (6.5-11.4)	15.8 (15.5-16.1)	
51-60	15.2 (12.6-18.3)	16.1 (15.8-16.4)	
≥ 61	72.7 (69.0-76.1)	28.7 (28.2-29.2)	
Race/ethnicity			
Non-Hispanic White	95.9 (94.1-97.2)	61.9 (60.5-63.3)	**<.0001**
Non-Hispanic Black	0.7 (0.3-1.5)	11.9 (11.1-12.7)	
Hispanic	2.0 (1.1-3.5)	17.4 (16.2-18.6)	
Non-Hispanic Asian	0.3 (0.1-1.1)	6.1 (5.7-6.6)	
Non-Hispanic other	1.2 (0.6-2.2)	2.7 (2.3-3.1)	
Region			
Northeast	15.3 (12.3-19.0)	17.5 (16.5-18.6)	.6
Midwest	21.0 (18.0-24.5)	20.7 (19.6-21.8)	
South	39.7 (35.7-43.9)	38.1 (36.5-39.8)	
West	23.9 (20.4-27.8)	23.7 (22.2-25.2)	
BMI‡			
Underweight	1.7 (1.0-2.8)	1.7 (1.6-1.8)	.2
Healthy weight	30.7 (27.4-34.3)	31.1 (30.6-31.6)	
Overweight	37.3 (33.9-40.8)	33.9 (33.5-34.3)	
Obese	30.3 (27.0-33.8)	33.3 (32.8-33.8)	

Values that are statistically significant (2-side *P* value ≤ .05) are in bold.

*BMI*, Body mass index; *CI*, confidence interval; *N/A*, not applicable; *NHIS*, National Health Interview Survey.

∗Weighted percentage was calculated using NHIS survey design parameters.

† Melanoma of skin status was assessed by the question “Have you ever been told by a doctor or other health professional that you had skin melanoma?”

‡ Underweight is a BMI < 18.5; healthy weight is a BMI 18.5 to < 25; overweight is a BMI ≥ 25 to < 30; obese is a BMI ≥ 30.

**Table II:** Association between melanoma of skin and various cancers in adults aged 18 years and more

Cancer	Total cases (unweighted)	Unadjusted OR (95% CI)	*P* value	Adjusted OR (95% CI)∗	*P* value	Bonferroni *P* value
Bladder	328	6.02 (3.47-10.42)	**<.001**	2.31 (1.31-4.06)	**.004**	.06
Blood	74	3.04 (0.68-13.65)	.2	1.55 (0.34-7.07)	.6	1.0
Brain	72	1.23 (0.17-8.93)	.8	1.01 (0.14-7.29)	1.0	1.0
Breast	2094	2.66 (1.87-3.77)	**<.001**	1.49 (1.03-2.17)	**.03**	.5
Colon and rectal	610	3.42 (1.97-5.93)	**<.001**	1.65 (0.95-2.87)	.08	1.0
Esophageal	41	4.83 (0.66-35.30)	.1	1.77 (0.24-12.89)	.6	1.0
Larynx	17	24.24 (6.34-92.72)	**<.001**	9.97 (2.64-37.58)	**.001**	**.02**
Leukemia	164	3.01 (1.34-6.79)	**.008**	1.45 (0.60-3.46)	.4	1.0
Liver	60	7.44 (2.41-22.90)	**<.001**	3.79 (1.25-11.51)	**.02**	.3
Lung	376	3.19 (1.48-6.85)	**.003**	1.38 (0.64-2.98)	.4	1.0
Lymphoma	338	3.24 (1.60-6.55)	**.001**	1.88 (0.93-3.82)	.08	1.0
Mouth	49	2.44 (0.33-17.86)	.4	1.22 (0.17-8.92)	.8	1.0
Stomach	62	4.18 (0.89-19.73)	.07	2.33 (0.50-10.83)	.3	1.0
Throat	106	1.71 (0.39-7.51)	.5	0.73 (0.17-3.20)	.7	1.0
Thyroid	344	1.15 (0.50-2.67)	.7	0.86 (0.37-2.01)	.7	1.0
Head and neck	171	3.73 (1.57-8.88)	**.003**	1.66 (0.70-3.93)	.2	1.0

Values that are statistically significant (2-side *P* value ≤ .05) are in bold.

*CI*, Confidence interval; *OR*, odds ratio.

∗ Logistic regression models adjusted for sex, cigarette smoking, and age.

Previous research has highlighted shared genetic variants across bladder, breast, laryngeal, and liver malignancies and cutaneous melanoma.^3^, ^4^, ^5^ Specifically, elevated expression of the melanoma-associated antigen-A gene has been linked to hepatocellular carcinoma, more aggressive melanoma, breast, and bladder cancers and worse survival outcomes in breast cancer and laryngeal squamous cell carcinoma.^3^ Additionally, BAP1 germline variants are found in melanoma, bladder, and breast cancer, while midnolin gene expression is detected in melanoma, liver, and breast cancers.^4^^,^^5^ These shared genes may partially explain significant associations between melanoma and bladder, breast, liver, and laryngeal cancer. However, while the genetic overlap may point to a link between the cancers, it does not indicate a causative relationship. Therefore, it is important for clinicians to evaluate melanoma patients for future melanomas while also considering other primary malignancies.

To further advance our understanding of these associations, future research should investigate shared genetic susceptibilities and nongenetic contributors such as lifestyle, dietary patterns, and environmental exposures. Examining modifiable factors may help inform prevention strategies and elucidate shared oncogenic pathways. As melanoma incidence continues to rise, ongoing research will be critical to determine whether demographic differences observed reflect true variations in population risk or underdiagnosis driven by under-recognition or healthcare disparities. Limitations of this study include small sample size, self-reported data and missclassfications, survivorship and detection bias, lack of clinical details and cancer staging, and preclusion of temporarily and causality reported from a cross-sectional survey database.

## Conflicts of interest

Dr Tung is or has been a clinical trials investigator, consultant, or speaker for AbbVie, Alumis, Amgen, Apogee, Arcutis, Boehringer Ingelheim, BMS, Castle Biosciences, Eli Lilly, Galderma, Incyte, Johnson & Johnson, Novartis, Pfizer, Regeneron, Sanofi, Sun Pharmaceutical, Takeda, and UCB. Aya Youssef, Dr Kim, and Dr Smith have no conflicts of interest to declare.
